# Endovascular Treatment of Late Thoracic Aortic Aneurysms after Surgical Repair of Congenital Aortic Coarctation in Childhood

**DOI:** 10.1371/journal.pone.0083601

**Published:** 2013-12-26

**Authors:** Robert Juszkat, Bartlomiej Perek, Bartosz Zabicki, Olga Trojnarska, Marek Jemielity, Ryszard Staniszewski, Wiesław Smoczyk, Fryderyk Pukacki

**Affiliations:** 1 Department of Clinical and Interventional Radiology, Poznan University of Medical Sciences, Poznan, Poland; 2 Department of Cardiac Surgery and Transplantology, Poznan University of Medical Sciences, Poznan, Poland; 3 Department of Cardiology, Poznan University of Medical Sciences, Poznan, Poland; 4 Department of General and Vascular Surgery, Poznan University of Medical Sciences, Poznan, Poland; 5 Department of Pediatric Radiology, Poznan University of Medical Sciences, Poznan, Poland; Scuola Superiore Sant'Anna, Italy

## Abstract

**Background:**

In some patients, local surgery-related complications are diagnosed many years after surgery for aortic coarctation. The purposes of this study were: (1) to systematically evaluate asymptomatic adults after Dacron patch repair in childhood, (2) to estimate the formation rate of secondary thoracic aortic aneurysms (TAAs) and (3) to assess outcomes after intravascular treatment for TAAs.

**Methods:**

This study involved 37 asymptomatic patients (26 female and 11 male) who underwent surgical repair of aortic coarctation in the childhood. After they had reached adolescence, patients with secondary TAAs were referred to endovascular repair.

**Results:**

Follow-up studies revealed TAA in seven cases (19%) (including six with the gothic type of the aortic arch) and mild recoarctation in other six (16%). Six of the TAA patients were treated with stentgrafts, but one refused to undergo an endovascular procedure. In three cases, stengrafts covered the left subclavian artery (LSA), in another the graft was implanted distally to the LSA. In two individuals, elective hybrid procedures were performed with surgical bypass to the supraaortic arteries followed by stengraft implantation. All subjects survived the secondary procedures. One patient developed type Ia endoleak after stentgraft implantation that was eventually treated with a debranching procedure.

**Conclusions:**

The long-term course of clinically asymptomatic patients after coarctation patch repair is not uncommonly complicated by formation of TAAs (particularly in individuals with the gothic pattern of the aortic arch) that can be treated effectively with stentgrafts. However, in some patients hybrid procedures may be necessary.

## Introduction

Aortic coarctation is a congenital stenosis at the level of the aortic isthmus, located between the left subclavian artery (LSA) and the arterial ligament. It accounts for 5–10% of the congenital cardiac malformations that are treated surgically [1], [2]. Despite significant progress in the surgical management of the aortic coarctation, surgery is still associated with potential complications during the follow-up period that include hypertension, premature coronary artery disease and cerebrovascular disease [3]. Moreover not uncommonly, late local complications such as peri-anastomotic aneurysms or recurrent coarctations are diagnosed even in asymptomatic patients [2]–[5]. It is generally accepted that TAA requires repair, otherwise it can rupture and cause sudden death [6]. Repeated open surgery carries significant mortality and morbidity, including bleeding and adverse neurological events, such as paralysis of the recurrent nerve or paraplegia [7], [8]. Nowadays, endovascular repair with stentgrafts seems to be a desirable therapeutic option due to its minimally invasive approach and favorable outcomes [9], [10]. All symptomatic late local complications are unquestionable indications for urgent interventions [10]. However, questions regarding the optimal timing of surgery for asymptomatic patients remains unresolved. In such a clinical scenario, we must balance the risk of any invasive procedures and the possible benefits.

The purposes of this study were: (1) to systematically evaluate asymptomatic adults after Dacron patch repair in childhood for isolated aortic coarctation, (2) to estimate the formation rate of secondary thoracic aortic aneurysms (TAAs) and (3) to assess outcomes after intravascular treatment for TAAs.

## Materials and Methods

### Study population

This observational prospective study involved 37 patients (26 female and 11 male) (Table 1) who underwent surgical correction (Dacron patch aortoplasty) via a left thoracotomy in childhood. All patients were operated on by one experienced team in a single cardiac surgical center. They were 8.1±4.9 years old at the time of primary surgery. This follow-up study was initiated after patients had reached adulthood.

**Table 1:** Selected demographic and clinical characteristics of patients.

	N = 37
Gender [M/F]	26/11
Age^a^ [years ± sd (range)]	31.1±5.4 (25–38)
Age (primary surgery) [years ± sd (range)]	8.1±4.9 (1–14)
Time: primary surgery to secondary aneurysm detection [years ± sd (range)]	20.6±1.9 (18–24)
Total follow up period^b^ [years ± sd (range)]	24.4±2.4 (19–27)
Patients without abnormalities [n(%)]	24 (64.9%)
Patients with thoracic aortic stenosis [n(%)]	6 (16.2%)
Patients with secondary aortic aneurysm formation [n(%)]	7 (18.9%)

^a^ age at the last follow up examination (ie. after stentgraft deployment)

^b^ includes time between the primary and repeat procedures, and the follow up period after stentgraft implantation.

Patients who required repeat procedures (either surgical or endovascular) due to any clinical symptoms of the late complications, such as pain, rupture, recoarctation with severe hypertension or cerebrovascular events, were excluded from the study.

### Ethics statement

The Institutional Review Board at the Poznan University of Medical Sciences approved the study protocol and informed written consent was obtained from each study participant.

### CTA and echocardiographic follow-up after primary surgery

During the follow-up period that lasted from 1993 to 2012 and was completed by 100% of the patients, computed tomography angiography (CTA) was performed systematically (usually every two or three periods). The study was done in two series: a regular scan followed by a contrast enhanced scan after intravenous infusion of 50–80 ml of Iomeprol 400 (Iomeron 400, Bracco-ALTANA Pharma GmbH, Italy) at a rate of 4 ml/sec. Before 2008, CTA was performed using a four-row MDCT (Multidetector Computed Tomography) (Toshiba Aquillion, Japan); a 64-row MDCT (Light Speed VCT, GE Medical System, Milwaukee, USA) was used beginning in June 2008. The cross-section images, multiplanar and volume reconstructions were evaluated. In CTA, attention was paid not only to detect any aortic pathologies (stenosis, aneurysm etc), but also to assess the pattern of the aortic arch following its repair for coarctation according to classification of Ou et al [11].

All patients also underwent echocardiographic (M+2D+Doppler) follow-up with the use of a transthoracic probe. More frequent studies (usually once every six months) were carried out in patients with a co-existing biscuspid aortic valve (BAV) because they are at particularly high risk for the formation of ascending aorta aneurysm and dissection [12].

### Invasive procedures of TTA treatment

Detection of TAA (i.e., exceeding 5.0 cm in diameter) in CTA was an indication for the invasive treatment. One of the patients diagnosed with TAA below the LSA level refused intervention. All implantation procedures of the stentgraft were performed in the angiography suite equipped with an Integris Allura (Phillips, The Netherlands) device since 2007. Endovascular procedures were carried out under spinal anesthesia. Digital subtraction angiography (DSA) was carried out in three different projections to accurately visualize the aortic arch and its branches, the aneurysmal sac and its relation to the LSA orifice. The left anterior oblique view was usually optimal to control the deployment of the stentgraft. The thoracic stentgrafts (Zenith, Cook, USA) were inserted through the surgically exposed right common femoral artery and were advanced to the aortic arch and the descending thoracic aorta using a stiff Amplatz guidewire. The stentgraft diameter and length were selected to exceed the diameter of the proximal landing zone by approximately 15% and to cover all segments of the aneurysmal aorta with a mandatory margin of at least 20 mm both proximally and distally.

### Post-procedural follow-up

In patients with LSA covered by proximal segment of stentgraft, regular Doppler ultrasound studies were performed, the first one usually a few hours after the intravascular procedure. Moreover, all patients who underwent stentgraft implantation were evaluated with CTA prior to discharge (usually on fifth to seventh post-procedural day), and then six months after the intervention. The post-discharge clinical and CTA follow-up were done on a regular basis every 12 months.

### Data management

First, the continuous variables were checked for normality using the Shapiro-Wilk W test. The data with a normal distribution (e.g., age, years of follow-up after primary surgery) were expressed as the mean with standard deviation. For non-parametric data (e.g., follow-up period after endovascular procedures) the median and the range are presented. Calculations were carried out using Statistica 9.0 (StatSoft, Tulsa, USA).

## Results

### CTA and echocardiographic follow-up

Based on follow-up CTA, three types of aortic arches were distinguished following surgical repair of coarctation (romanesque (n = 20), gothic (n = 9), and crenel (n = 8)). CTA follow-up examinations revealed no pathologies of the thoracic aorta in 24 patients (65%) who underwent Dacron patch aortoplasty in childhood. In six examined individuals (16%), mild stenosis was detected in the aortic isthmus with a mean diameter of 18.1±3.1 mm (ranging from 14 to 21 mm). In another seven studied subjects (19%) (three women and four men), aneurysms of the descending aorta with a mean diameter of 56.9±7.1 mm were found (see Table 2). In the majority of them (six cases) the aortic arch was the gothic type and only one was romanesque. The mean time period between primary surgery for aortic coarctation and the diagnosis of TAA was 20.6±1.9 years. In three cases, the aneurysms involved the LSA orifices and in two other patients the aneurysmal sac was located 10 to 20 mm distal to the LSA origin (including the only one with the romanesque pattern in the aneurysm group). In two consecutive patients, TAAs comprised the LSA and the origin of the left common carotid artery.

**Table 2:** Characteristics of patients with the secondary thoracic aortic aneurysm.

No	Age at primary surgery and aneurysm detection [years]	Aortic valve type*	Diameter of aortic root, ascending aorta and proximal arch [mm], aortic arch pattern**	Size of aneurysm [mm]	Method of treatment	Adverse events
1	14, 34	TAV	34, 32, 29, G	58	Stentgraft covering the LSA	No
2	11, 35	TAV	32, 30, 33, G	54	stentgraft covering the LSA	No
3	10, 31	BAV	34, 28, 30, R	53	stentgraft below the LSA	No
4	12, 31	TAV	31, 33, 32, G	68	stentgraft covering the LSA, LSA embolization, failed open surgery, debranching procedure (a bifurcated prosthesis and stemtgraft implantation in the aortic arch)	type Ia endoleak
5	1, 22	TAV	28, 31, 30, G	51	Refusal of endovascular procedure, medical treatment	-
6	6, 24	TAV	32, 30, 30, G	64	Hybrid procedure (bypass to aortic branches, stentgraft across the aortic arch)	No
7	3, 24	TAV	34, 33, 32, G	54	Hybrid procedure (bypass to aortic branches, stentgraft across the aortic arch)	No

aortic valve types: BAV  =  bicuspid aortic valve, TAV  =  tricuspid aortic valve;

aortic arch patterns: G  =  gothic, R  =  Romanesque.

In seven (18.9%) individuals, BAV was diagnosed, although its function was assessed as normal and nobody required any intervention on the aortic valve. Throughout the follow-up period, no patients developed an ascending aorta aneurysm or dissection that required surgical intervention.

### Invasive treatment of TAAs

All patients with TAAs diagnosed by CTA were qualified for endovascular therapy. One female patient with an aneurysm of borderline indication, the smallest one in our series (51 mm in diameter), refused any intervention due to a lack of clinical symptoms and a full sense of health. She strictly followed our recommendations regarding oral medications (e.g. beta-blockers, antihypertensive agents) and lifestyle. Since the time of diagnosis she has undergone regular clinical and CTA evaluation.

**Figure 1:**
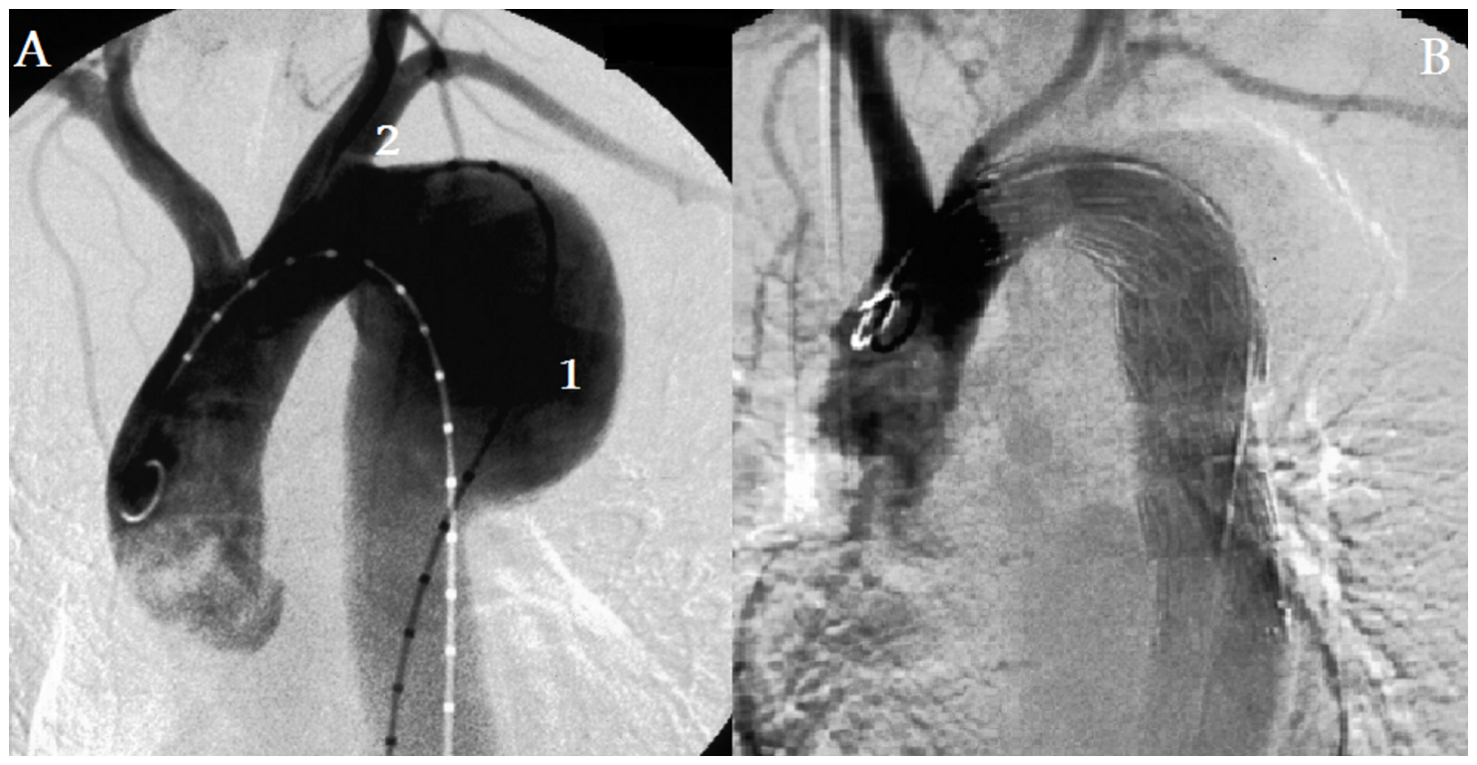
Stentgraft covering the LSA. Before (A) and after (B) stentgraft implantation. Aortic aneurysm (1) was located close to the LSA (2). An implanted stentgraft excluded the aneurysmal sac but covered the LSA (2) origin from the distal aortic arch. (Table 2, patient No 1)

**Figure 2:**
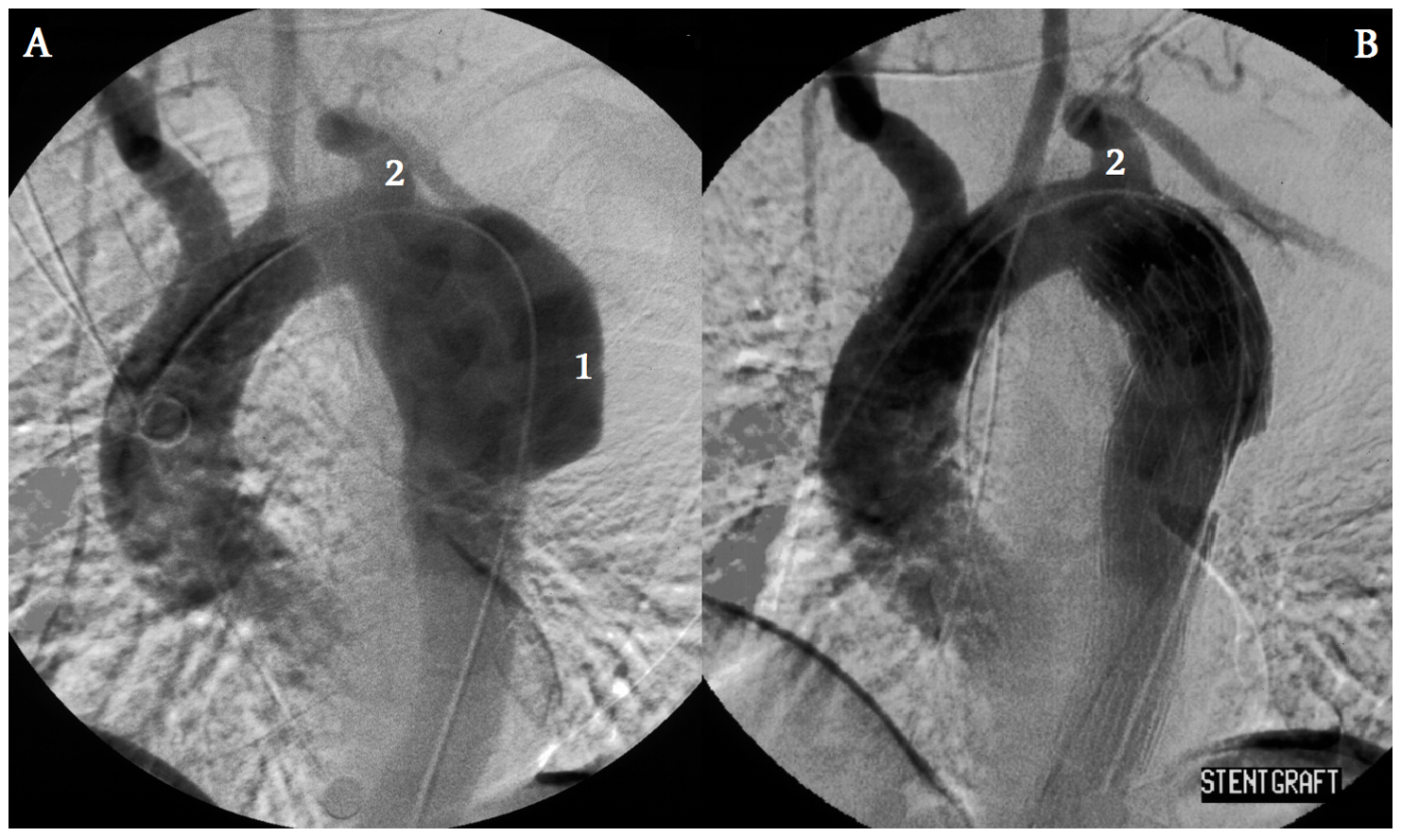
Stentgraft implanted below the LSA. Before (A) and after (B) stentgraft implantation. Aortic aneurysm (1) was located enough distally to the LSA (2) origin to implant a stentgraft without compromising flow in the LSA. (Table 2, patient No 3)

**Figure 3:**
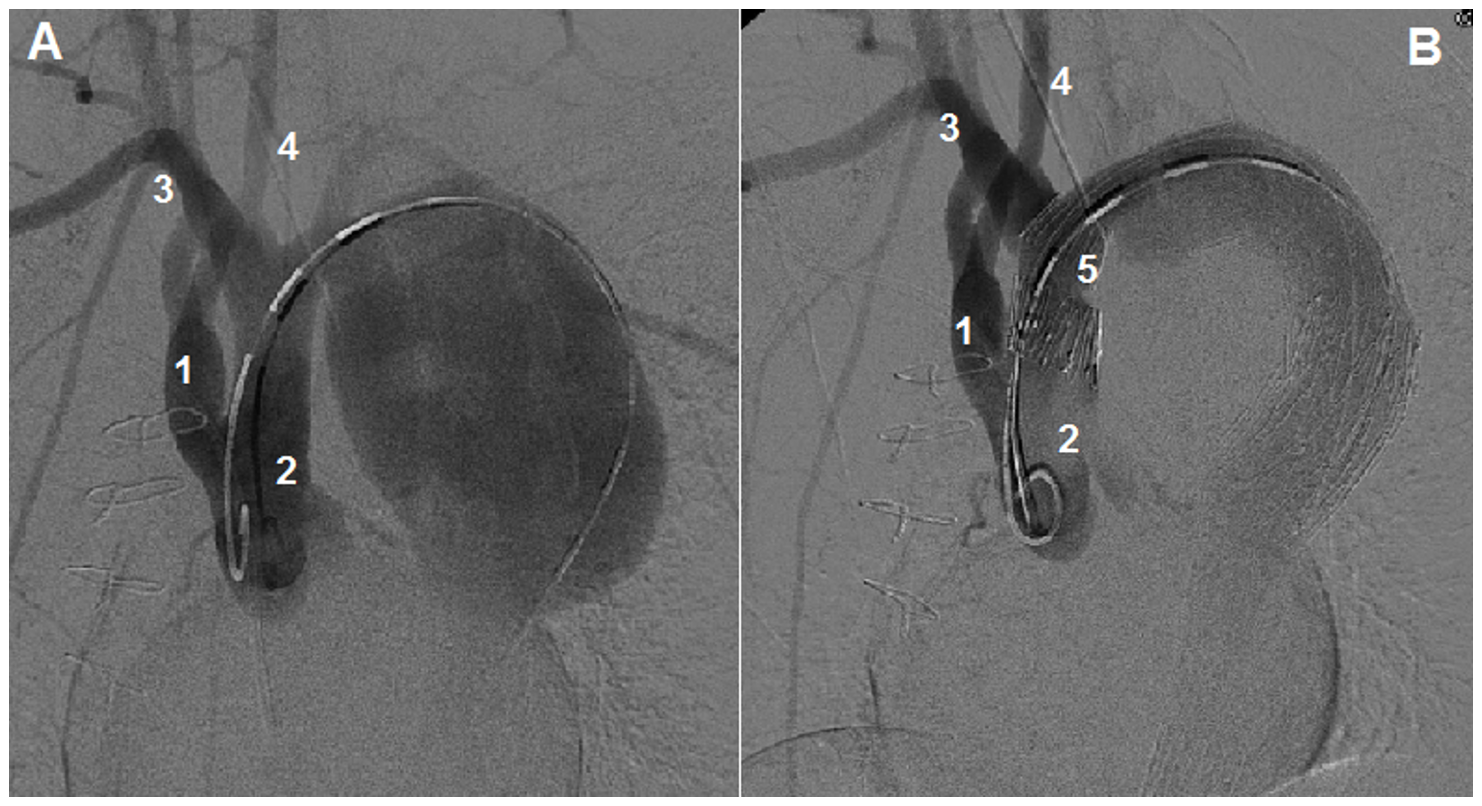
Hybrid method of aneurysm treatment. A hybrid approach for treatment of the aneurysm that involved the distal part of the aortic arch. Before (A) and after (B) stentgraft deployment. During the first surgical stage, a bifurcated prosthesis (1) was inserted between the ascending aorta (2) and the brachiocephalic trunk (3) and the left common carotid artery (4). In the second intravascular stage, a stentgraft (5) was implanted across the aortic arch. (Table 2, patient No 6)

Six individuals with TAA underwent implantation of the stentgraft. In all patients, due to the relatively small length of the aneurysmal sac, single stentgrafts were enough to cover the pathological aortic segments. Neither tapered nor customized stentgrafts were implanted. Having in mind the exact obligatory margin in the proximal landing zone, stentgrafts were implanted exactly below the left common carotid artery in three cases. The LSA was covered with the grafts (Fig. 1). Only in one case was the landing zone below the LSA origin (Fig. 2). Two other patients underwent open surgery prior to stentgraft implantation because the distal part of the aortic arch was involved in the TAA. The operations were performed via median sternotomy. After mobilization of the left brachiocephalic vein, all branches of the aortic arch were dissected free and heparin was administrated at a dose of 150 IU/kg. Then bifurcated vascular prostheses were attached in an end-to-side fashion with 4-0 monofilament suture to the middle segment of the partially clamped ascending aorta. Afterwards, one leg of the bifurcated prosthesis was anastomosed end-to-side with the brachocepalic trunk and the second one with the left carotid artery. The final step of the surgery was ligation of the aortic arch branches to prevent backflow into the proximal segments of the bypassed arteries to minimize the risk of any leakage around the stentgrafts implanted a few days later. Owing to surgery, the proximal landing zone was extended and enabled the implantation of the stentgraft across the aortic arch (Fig. 3).

### Post-procedural outcome

All patients survived the invasive procedures. There were no periprocedural complications and in all cases the pre-discharge control CTA scans revealed complete exclusion of the aneurysm. None of the patients developed central neurological events. Special attention was paid to patients with a covered LSA. The post-procedural Doppler ultrasound studies of 3 patients with the covered LSA showed subclavian steal syndrome (Type 1, Grade 3). However, in spite of reversed flow in the vertebral artery, all patients were clinically asymptomatic, so they did not require a carotid-to-subclavian bypass. One patient with the orifice of the LSA covered by the proximal segment of the stentgraft reported pain 48 hours after the procedure in the occipital area. It was relieved with non-steroid anti-inflammatory drugs.

**Figure 4:**
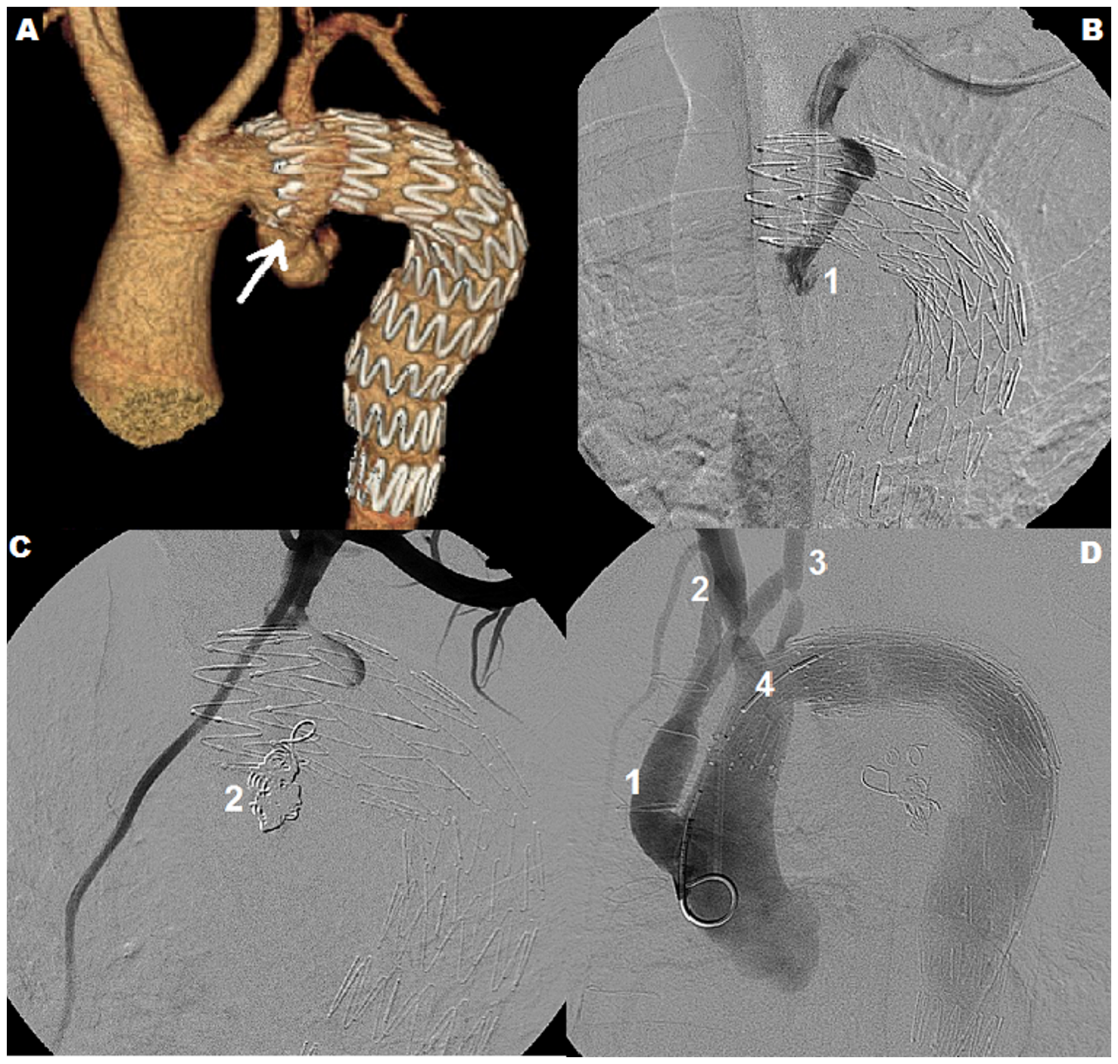
Treatement of stentgraft endoleak. Type Ia endoleak (↑) 6 months after the stentgraft implantation (A). Status with type Ia endoleak (1) before (B) and after (C) treatment with the Tornado coils (2). A final result of the debranching procedure (D) with a bifurcated Dacron prosthesis (1) to the brachiocephalic trunk (2) and the left common carotid artery (3) followed by implantation of the stentgraft (4) that covered all the native branches of the aortic arch. (Table 2, patient No 4)

All patients with secondary TAAs completed a follow-up period that lasted from 14 to 49 months (median 26 months). In one patient, at the six-month CTA follow-up examination, a type Ia endoleak was diagnosed. In this case, the LSA was the outflow vessel for the aneurysm (Fig. 4a and 4b). Thus the patient underwent embolization of the aneurysmal sack and the LSA origin using six Tornado coils (Cook, USA). The coils were delivered intravascularly through the DAV catheter (Cook, USA) inserted via the left axillary artery. A control angiography at the completion of the procedure revealed no persisting endoleak to the sack of the aneurysm (Fig. 4c). After another three months, continuous flow in the aneurysm to outside the graft was detected again. A decision to treat the patient surgically from median sternotomy was undertaken. The site of leakage from the aortic arch to the aneurismal sac was closed successfully using interrupted sutures with Teflon patches. Unfortunately, the good result was only temporary. Although the patient remained asymptomatic, three months after surgery, a leak close to the proximal landing zone of the stentgraft was visualized again. The next step was reoperation as a part of the combined debranching procedure. A bifurcated Dacron prosthesis that connected the ascending aorta to the brachiocephalic trunk and the left common carotid artery was implanted and a stentgraft that covered all vessels of the aortic arch was deployed. The latter procedure successfully completed treatment of this particular TAA case (Fig 4d). Further follow-up (26 months), both clinical and angiographic, was uneventful.

The last two patients who underwent elective hybrid procedures did not present any complications after the surgical and endovascular stages of combined therapy. The follow-up CTA scans revealed no flow within the sac of the TAA and the patent bypasses to either the brachiocephalic trunk or the common carotid arteries were observed. Upon physical examination, patients did not present any neurological deficit.

## Discussion

Surgical repair of an aortic coarctation in childhood is a relatively safe procedure that has been performed for many years [2]. However, some years ago, minimally invasive balloon percutaneous angioplasty (PTA) was proposed as a promising alternative to open surgery [1]. Restenoses and the formation of secondary aneurysms at the site of the previous surgical manipulations are the most frequent late complications [1], [13]. The prevalence of secondary aneurysms varies significantly between published reports, from 2 to 33% [4], [8]. Thus, the almost 20% in our study is within the range of the findings in the aforementioned publications. There are some hypotheses regarding the formation of the postsurgical aneurysms. Some of them stress possible mechanical and biological insufficiency of reunion between the native vessel and the implanted prosthetic patch, while others suggest a role of the intraoperative injury to the vasa vasorum and degeneration of the tunica media [14]–[17]. Moreover, we showed that the majority of patients (85.7%) with secondary aneurysm had the gothic type of aortic arch following surgical repair in childhood. Otherwise, in the group without an aneurysm, the prevalence of this type was markedly lower (10%). This finding is consistent with previous reports showing that the gothic pattern of aortic arch is associated with significant morbidity in long-term follow-up, due to blood flow disorganization in the descending aorta even in the absence of a recurrent coarctation [11], [18], [19].

According to the guidelines published in 2010 for the management with thoracic aortic disease, patients with postoperative aneurysm should be treated if the aneurysm of the descending thoracic aorta exceeds 5.5 cm and endovascular stent grafting should be strongly considered when feasible [20]. However, in the aforementioned recommendations there is no information about biological variability with respect to weight, height, body surface area or body mass index. In our group of participants, all patients were young and fit. Moreover, keeping in mind the possible pathogenic factors, such as impaired connective tissue, and the fact that elective invasive procedures are much safer than emergent ones [10], we decided to perform the endovascular procedures earlier. Moreover, a conservative treatment of a secondary aortic aneurysm is unpredictable. For example, Knyshov et al. reported a 100% rate of rupture at 15 years whereas Cohen et al. observed a less than 7% mortality rate related to aortic complications [6], [21].

The surgical management of TAA is a demanding procedure with a relatively high risk of mortality and significant morbidity, particularly in patients who require re-thoracotomy for aortic reconstruction [22]. However, some experienced surgeons still find surgical repair to be an effective therapeutic option, competitive to endovascular procedures. Although endovascular repair of the aneurysms was initiated by Dotter in 1969, the first implantation of a stengraft to treat an aneurysm of the descending aorta was reported in 1991 [23], [24]. The first reports dealing with endovascular repairs of TAA that complicated the late follow-up period after surgical correction of coarctation appeared a few years ago [9], [25], [26]. These patients are a particular group because in some cases, the anatomy is not clear (even in CTA), the mechanical properties of the native aorta and the artificial patch are different (which makes endovascular procedures less safer, and technically more demanding), the aortic arch may be hypoplastic or the aneurysm may be preceded by restenosis that requires balloon dilatation [27]. Thus, more experienced interventional radiologists, cardiac and vascular surgeons should be involved in the treatment. Not uncommonly, combined procedures must be performed [16], [28]. In our series, four cases were treated successfully with single stentgrafts. Another three required more complex interventions that included a hybrid approach. Even if the post-surgical aneurysm involves the distal aortic arch, endovascular therapy with stentgraft implantation is a crucial step. The conventional open surgical corrections of aortic arch aneurysms usually require cardiopulmonary bypass and deep hypothermic circulatory arrest or selective cerebral perfusion. As a consequence, they are associated with high mortality and morbidity. Thus, hybrid treatment for aortic arch aneurysms has been proposed as an intriguing alternative to the conventional operation. It combines the surgical debranching procedure with interposition of the graft between the ascending aorta and the main branches of the aortic arch followed by retrograde stentgraft deployment [28], [29]. The safety and efficiency of this technique were confirmed in our study.

The introduction of a less-invasive method to treat TAAs secondary to coarctation repair significantly decreased the mortality rate to minimal. In our series, nobody died, even in the group of individuals who underwent the combined hybrid procedure. The most serious local complications after stentgraft deployment were endoleaks. They accounted for approximately 5% of patients undergoing endovascular treatment [30]. In our case, an endoleak required not only the intravascular but also surgical re-intervention. On the other hand, the intravascular methods enabled us to reduce the prevalence of systemic (neurological, cardiac, respiratory) dysfunction and ischemic injury to the visceral organs [9], [27]. Despite of use of a large volume of contrast medium, none of the patients in our group developed acute renal failure. Our findings support earlier reports dealing with morbidity and mortality related to the treatment of the TAAs secondary to coarctation repair many years before [9], [26]–[28].

The authors are aware of possible complications that can appear many years after stentgraft implantation, such as aneurysm formation/progression in the untreated aortic segments, rupture of the aneurysm or stentgraft migration [31]. The patients treated intravascularly by our team were relatively young (a long period of future long-term follow-up) and with history of anatomic congential malformation of the aorta (usually associated with defective tissue). Some of them had also BAV, which is an accepted risk factor for the development of an aortic aneurysm or dissection [12]. Each time before the intravascular procedure, we balanced the risks and benefits related to therapeutic management and we had to choose between open surgery and endovascular therapy. Moreover, we discussed this issue with patients and all of them wanted to avoid repeat surgery (due to bad memories of the primary surgery since four of them were adolescents at that time) even if future surgery for progression of the aneurysm in the untreated aortic segments would be more risky and technically demanding. We decided to perform hybrid procedures only if they were really necessary due to the location of the aneurysm. Although, debranching procedures themselves are relatively safe for an experienced team of cardiac and vascular surgeons, they are invasive and performed via median sternotomy. Additionally, side implantation of the bifurcated prosthesis to the ascending aorta seriously complicates any future cardiac surgical operations with the use of cardio-pulmonary bypass, such as aortic valve replacement with/without aortic root replacement (for BAV) or coronary artery bypass surgery. In our opinion, it is less challenging to perform a surgical procedure of arch anastomosis once a stent is placed. Thus, we always tried to choose the less invasive method, but every time the final decision regarding the therapeutic option was considered very carefully and individually.

Finally, we must be aware of the limitations of the present and previous studies that assessed the safety and outcomes of intravascular options. Nowadays, there is insufficient data in this field and the follow-up period after endovascular repair is too short to make final conclusions. More patients should be treated with stentgrafts to assess the efficacy of this promising less-invasive alternative to open re-do surgery after coarctation repair in childhood.

## Conclusions

Our study indicates that a long-term course of clinically asymptomatic patients after coarctation patch repair in childhood is not uncommon (particularly in individuals with a gothic pattern of the aortic arch) complicated by the formation of TAAs that can be treated effectively with stentgrafts. Due to the low mortality and morbidity rates, the endovascular option may be considered as a method of choice in the management of secondary TAAs. However, in some patients with involvement of the aortic arch, hybrid procedures may be necessary to achieve a successful outcome.
